# Cardiac function and atrial conduction time in morbid obesity: Insights from an echocardiographic case-control study

**DOI:** 10.21542/gcsp.2025.13

**Published:** 2025-02-28

**Authors:** Fatemeh Omidi, Soheila Sadeghi, Mohammad Javad Nasiri, Zeinab Ghaffari, Kiana Ghafourian, Masoud Hosain Panahi

**Affiliations:** 1 Prevention of cardiovascular disease research center, Shahid Beheshti University of Medical Sciences, Tehran, Iran; 2 Department of Internal Medicine, Imam Hossein Hospital, Shahid Beheshti University of Medical Sciences, Tehran, Iran

## Abstract

Background: Obesity, especially morbid obesity, is linked to increased cardiovascular risk, including potential abnormalities in atrial conduction and changes in cardiac structure. This study examines the impact of morbid obesity on atrial conduction time (ACT) and associated echocardiographic parameters.

Methods: This case-control study involved 100 patients from Imam Hossein Hospital, Tehran, including 50 with morbid obesity and 50 with normal BMI. Demographic, clinical, and echocardiographic data were collected. ACT was measured, and echocardiographic parameters including left atrial septum thickness, posterior wall thickness, and mitral valve peak early diastolic velocity (MV.PAV.1) were evaluated.

Results: The morbid obesity group exhibited significantly prolonged ACT (*p* = 0.007) compared to the normal BMI group, indicating impaired atrial conduction. Echocardiographic analysis revealed that left atrial septum thickness (0.90 ± 0.05 vs. 0.85 ± 0.07, *p* = 0.012), posterior wall thickness (0.84 ± 0.05 vs. 0.80 ± 0.03, *p* = 0.034), and MV.PAV.1 (73.68 ± 17.67 vs. 61.35 ± 14.44, *p* = 0.029) were significantly higher in the morbid obesity group compared to the normal BMI group.

Conclusions: Morbid obesity is associated with prolonged ACT and increased echocardiographic measurements of left atrial septum thickness, posterior wall thickness, and MV.PAV.1. These findings suggest that morbid obesity adversely affects atrial conduction and cardiac structure, highlighting the need for targeted cardiovascular risk management in obese patients.

## Introduction

Obesity is a well-known risk factor for various cardiovascular diseases, including hypertension, coronary artery disease, and heart failure^1–3^. Among its classifications, morbid obesity, defined as having a body mass index (BMI) of 35 or higher, poses an even higher risk for cardiovascular complications^4–7^. One of the significant yet less studied aspects of cardiovascular risk in morbid obesity is its impact on atrial conduction and cardiac structure^8–11^.

Atrial conduction time (ACT) is a critical parameter in assessing the electrical conduction system of the heart^12–14^. Prolonged ACT can be indicative of atrial conduction abnormalities, which are associated with an increased risk of atrial arrhythmias, such as atrial fibrillation^15–17^. Furthermore, obesity can lead to structural changes in the heart, including increased wall thickness and altered diastolic function, which may exacerbate these conduction abnormalities^18–21^.

While previous research has linked obesity to changes in heart function, there is still much we don’t fully understand about how severe obesity specifically impacts ACT^22–24^. Most studies have focused on broader electrical and structural changes in obesity without distinguishing between different levels of severity or examining key echocardiographic markers. Additionally, the connection between ACT and structural heart changes, such as atrial enlargement and myocardial remodeling, is not yet well defined^25,26^.

This study aims to explore the relationship between morbid obesity and ACT by comparing it with normal BMI and overweight groups. We also examine echocardiographic parameters, such as left atrial septum thickness, posterior wall thickness, and mitral valve peak early diastolic velocity, to provide a comprehensive assessment of how obesity influences cardiac structure and function.

## Methods

This case-control study was conducted on 100 patients (50 with obesity and 50 with normal weight) attending Imam Hossein Hospital in Tehran in the year 2024. Inclusion criteria consisted of adult patients (aged 18 years and older), while exclusion criteria included a history of heart disease and refusal to participate in the study.

### Sample Size

The sample size for this study was determined based on feasibility and the availability of patients at the study site. To ensure that our sample size was adequate to detect clinically meaningful differences, we conducted a post-hoc power analysis using G*Power (version 3.1.9.7) based on the observed effect sizes in our study.

### Definitions of study groups

• Group 1: Patients with morbid obesity (BMI ≥ 35) without history of heart disease.
• Group 2: Patients with overweight BMI (25–29.9) without history of heart disease.
• Group 3: Healthy individuals with normal BMI and no history of heart disease.

Following the acquisition of informed consent from the patients, demographic, clinical, and echocardiographic data for all study variables were extracted from patient histories and medical records. Diastolic cardiac function and ACT were assessed using transthoracic echocardiography with a PHILIPS EPIQ 7 device, conducted by a fellowship-trained echocardiographer. All data were collected and reported using a researcher-developed checklist, created by the study investigator (a specialist in internal medicine). The study was approved by the Ethics Committee of Shahid Beheshti University of Medical Sciences, Tehran, Iran (IR.SBMU.MSP.REC.1403.034).

### ACT measurement protocol

Atrial conduction time (ACT) was measured using tissue Doppler imaging from the apical four-chamber view. The interval from the onset of the P wave on the surface ECG to the peak of the A’ wave at the lateral mitral annulus was recorded as the ACT. All measurements were performed at a sweep speed of 100 mm/s to ensure precision.

### Observer variability

To assess measurement reliability, intra-observer and inter-observer variability were evaluated in a randomly selected subset of 20 patients. The same observer repeated the measurements two weeks apart to determine intra-observer variability, while a second independent echocardiographer performed the measurements to assess inter-observer variability. Variability was quantified using the intraclass correlation coefficient (ICC).

### Data analysis

Descriptive statistics (mean ± SD or median with interquartile range for continuous variables, and frequency with percentage for categorical variables) were calculated. Normality was tested with the Shapiro–Wilk test. Depending on the data distribution, omparisons were made using the Student’s *t*-test or Mann–Whitney U test for continuous variables, and the Chi-square test or Fisher’s exact test for categorical variables. Correlations were analyzed using Pearson’s or Spearman’s tests as appropriate. To adjust for confounders (age, sex, and underlying conditions), regression analyses were performed. A *p*-value of <0.05 was considered statistically significant, and all analyses were conducted using SPSS version 26.

## Results

### Demographic and Clinical Characteristics

The study included 100 patients, divided equally between those with obesity (BMI ≥ 35) and those with normal BMI. The results revealed notable differences in demographic and clinical characteristics across BMI categories (Table 1). The proportion of women was significantly higher in the obesity group (68.8%) compared to the overweight (45.8%) and normal weight (35.0%) groups (*P* = 0.037). Although age did not vary significantly between groups (*P* = 0.143), the overweight and obesity groups were slightly older on average. Weight and BMI differed significantly across groups (*P* < 0.001), with progressively higher values observed from normal weight to obesity. Abdominal circumference was also significantly larger in the obesity group (*P* < 0.001). Clinically, while the prevalence of hypertension and hyperlipidemia did not show significant differences (*P* = 0.07 and *P* = 0.742, respectively), systolic blood pressure was notably higher in the obesity group (*P* = 0.001). Total cholesterol levels were significantly elevated in the overweight and obesity groups (*P* < 0.001). Diastolic blood pressure and fasting blood sugar did not differ significantly across BMI categories.

**Table 1:** Demographic and clinical characteristics across BMI categories.

Characteristic	Normal (*n* = 20)	Overweight (*n* = 48)	Obesity (*n* = 32)	P-value
Sex Distribution				
Women	7 (35.0%)	22 (45.8%)	22 (68.8%)	0.037
Men	13 (65.0%)	26 (54.2%)	10 (31.3%)	
Age (years)	52.8 ± 8.43	57.29 ± 10.19	57.78 ± 8.94	0.143
Height (cm)	166.4 ± 10.5	163.6 ± 10.41	159.75 ± 11.62	0.087
Weight (kg)	63.35 ± 8.6	74.21 ± 10.24	84.53 ± 13.17	<0.001
BMI (kg/m^2^ )	22.81 ± 1.58	27.6 ± 1.29	35 ± 2.5	<0.001
Abdominal Circumference (cm)	87.35 ± 7.6	96.83 ± 8.14	108.84 ± 8.2	<0.001
Hypertension (HTN)				
No	15 (75.0%)	22 (45.8%)	15 (46.9%)	0.07
Yes	5 (25.0%)	26 (54.2%)	17 (53.1%)	
Hyperlipidemia (HLP)				
No	9 (45.0%)	19 (39.6%)	11 (34.4%)	0.742
Yes	11 (55.0%)	29 (60.4%)	21 (65.6%)	
Smoking Status				0.165
Non-smoker	14 (70.0%)	38 (79.2%)	29 (90.6%)	
Smoker	6 (30.0%)	10 (20.8%)	3 (9.4%)	
Total Cholesterol (mg/dL)	84.3 ± 8.18	117.23 ± 12.81	146.97 ± 14.15	<0.001
Systolic Blood Pressure (mmHg)	118.25 ± 15.1	129.23 ± 13.81	135.53 ± 16.64	0.001
Diastolic Blood Pressure (mmHg)	74.65 ± 9.47	79.15 ± 9.9	81.53 ± 10.48	0.059
Fasting Blood Sugar (mg/dL)	94.5 ± 8.41	98.5 ± 11.61	100.81 ± 14.59	0.194

### Atrial conduction time

Patients with morbid obesity had significantly prolonged ACT compared to those with normal BMI. The mean ACT in the morbid obesity group was notably higher (*p* = 0.007), indicating impaired atrial conduction in this population.

### Echocardiographic parameters

Septum: The left atrial septum thickness was significantly greater in the morbid obesity group (0.90 ± 0.05) compared to the normal BMI group (0.85 ± 0.07), with a *p*-value of 0.012 (Table 2).

**Table 2:** Echocardiographic parameters related to atrial conduction.

Parameter	Normal BMI (*n* = 50)	Morbid Obesity (*n* = 50)	*p*-value
Septum Thickness	0.85 ± 0.07	0.90 ± 0.05	0.012
Posterior Wall	0.80 ± 0.03	0.84 ± 0.05	0.034
MV.PAV.1	61.35 ± 14.44	73.68 ± 17.67	0.029

Posterior: The posterior wall thickness was also significantly higher in the morbid obesity group (0.84 ± 0.05) compared to the normal BMI group (0.80 ± 0.03), with a *p*-value of 0.034.

Mitral Valve Peak Early Diastolic Velocity (MV.PAV.1): The average value was higher in the morbid obesity group (73.68 ± 17.67) compared to the normal BMI group (61.35 ± 14.44), with a *p*-value of 0.029.

### Blood pressure parameters

Systolic Blood Pressure (SBP): Morbid obesity was associated with a significantly higher average SBP (131.75 ± 15.22) compared to those with normal BMI (118.25 ± 15.1), with a *p*-value of 0.001 (Table 3).

**Table 3:** Blood pressure measurements.

Measurement	Normal BMI (*n* = 50)	Morbid Obesity (*n* = 50)	*p*-value
SBP	118.25 ± 15.1	131.75 ± 15.22	0.001
DBP	74.65 ± 9.47	80.1 ± 10.14	0.032

Diastolic Blood Pressure (DBP): The DBP was also significantly higher in the morbid obesity group (80.1 ± 10.14) compared to the normal BMI group (74.65 ± 9.47), with a *p*-value of 0.032.

### Echocardiographic Parameters

Left Ventricular End-Diastolic Diameter (LVEDD): LVEDD was 4.83  ± 0.41 in the morbid obesity group compared to 4.88 ± 0.37 in the normal BMI group. The difference was not statistically significant (*p* = 0.575) (Table 4).

**Table 4:** Cardiac dimensions and function.

Parameter	Normal BMI (*n* = 50)	Morbid Obesity (*n* = 50)	*p*-value
LVEDD	4.88 ± 0.37	4.83 ± 0.41	0.575
LVEDS	3.12 ± 0.31	3.14 ± 0.35	0.802
EF	60.31 ± 4.9	59.21 ± 5.18	0.394
HR	67.25 ± 10.11	71.36 ± 10.6	0.121

Left Ventricular End-Systolic Diameter (LVEDS): LVEDS measured 3.14 ± 0.35 in the morbid obesity group and 3.12 ± 0.31 in the normal BMI group, with no significant difference (*p* = 0.802).

Ejection Fraction (EF): EF was 59.21 ± 5.18 in the morbid obesity group versus 60.31 ± 4.9 in the normal BMI group. This difference was not statistically significant (*p* = 0.394).

Heart Rate (HR): HR was 71.36 ± 10.6 in the morbid obesity group compared to 67.25 ± 10.11 in the normal BMI group, showing no significant difference (*p* = 0.121).

## Discussion

### Principal findings

This study investigated ACT and echocardiographic parameters among patients with different BMI categories. We found that patients with morbid obesity had significantly prolonged ACT compared to those with a normal BMI, with a *p*-value of 0.007. This suggests impaired atrial conduction in the morbid obesity group, which could increase the risk of atrial arrhythmias and other cardiovascular conditions. Additionally, echocardiographic parameters showed significant differences: left atrial septum thickness was greater in the morbid obesity group (0.90 ± 0.05) compared to the normal BMI group (0.85 ± 0.07), with a *p*-value of 0.012; posterior wall thickness was also increased in the morbid obesity group (0.84 ± 0.05 vs. 0.80 ± 0.03, *p* = 0.034); and Mitral Valve Peak Early Diastolic Velocity (MV.PAV.1) was higher in the morbid obesity group (73.68 ± 17.67) compared to the normal BMI group (61.35 ± 14.44), with a *p*-value of 0.029.

### Clinical implications

These findings have significant clinical implications for managing patients with morbid obesity. The prolonged ACT and echocardiographic abnormalities observed suggest a higher cardiovascular risk in this population. Specifically:

• Atrial Arrhythmias: The prolonged ACT in morbidly obese patients may indicate a higher risk of atrial fibrillation and other atrial arrhythmias. AF is a major risk factor for stroke and heart failure, and identifying patients with prolonged ACT could help in early intervention and prevention strategies^10,13,27,28^.
• Heart Structure and Function: Increased left atrial septum and posterior wall thickness, along with higher MV.PAV.1, suggest structural changes and potential impairment in diastolic function. These changes could lead to compromised cardiac function and should be monitored regularly in morbidly obese patients^29–38^.
• Targeted Monitoring and Intervention: Clinicians should consider regular echocardiographic assessments and ACT evaluations for morbidly obese patients. Early detection of atrial conduction abnormalities and structural changes can guide timely interventions, such as lifestyle modifications, pharmacological treatments, or surgical options, to mitigate cardiovascular risks^39,40^.
• Personalized Management Plans: The data highlights the need for personalized management plans that address not only weight reduction but also cardiovascular health. Comprehensive strategies, including lifestyle modifications like diet and physical activity, weight management, cardiovascular monitoring, and the potential use of medications to address atrial conduction issues, could improve overall patient outcomes. Tailoring interventions to individual patient needs ensures a holistic approach to managing the health risks associated with morbid obesity^41,42^.

## Limitations

This study has several limitations. The sample size of 100 patients may limit the generalizability of our findings, and the single-center design could introduce selection bias. Additionally, the significantly different gender distribution between groups (68.8% women in the obesity group vs. 35% in the normal BMI group) may act as a confounder. We also did not account for the impact of medications affecting cardiac function, nor did we assess physical activity levels and other lifestyle factors, which could influence atrial conduction and echocardiographic parameters. Future studies should consider these variables to provide a more comprehensive understanding of the relationship between obesity and cardiac function.

## Conclusions

This study highlights the association between morbid obesity and prolonged atrial conduction time, along with changes in echocardiographic parameters. These findings emphasize the increased cardiovascular risk in morbidly obese individuals and the importance of incorporating regular cardiac evaluations into their management plans. Future research should focus on longitudinal studies to further elucidate the impact of obesity on atrial conduction and cardiac function, and to develop targeted interventions to address these risks.

## Competing Interests

None.

## Data availability

The data used to support the findings of this study are included within the article.

## Author contribution

All author contributed equally to this work.
